# On the Stabilizing Effect of Aspartate and Glutamate and Its Counteraction by Common Denaturants

**DOI:** 10.3390/ijms25179360

**Published:** 2024-08-29

**Authors:** Guido Izzi, Marco Campanile, Pompea Del Vecchio, Giuseppe Graziano

**Affiliations:** 1 Institute of Biostructure and Bioimaging, National Research Council, Via P. Castellino, 80131 Naples, Italy; guidoizzi90@gmail.com; 2 Department of Chemical Sciences, University of Naples Federico II, Via Cintia, 80126 Naples, Italy; marco.campanile@unina.it (M.C.); pompea.delvecchio@unina.it (P.D.V.); 3 Department of Science and Technology, University of Sannio, Via F. De Sanctis, 82100 Benevento, Italy

**Keywords:** aspartate, glutamate, protein conformational stability, denaturants, counteraction

## Abstract

By performing differential scanning calorimetry(DSC) measurements on RNase A, we studied the stabilization provided by the addition of potassium aspartate(KAsp) or potassium glutamate (KGlu) and found that it leads to a significant increase in the denaturation temperature of the protein. The stabilization proves to be mainly entropic in origin. A counteraction of the stabilization provided by KAsp or KGlu is obtained by adding common denaturants such as urea, guanidinium chloride, or guanidinium thiocyanate. A rationalization of the experimental data is devised on the basis of a theoretical approach developed by one of the authors. The main contribution to the conformational stability of globular proteins comes from the gain in translational entropy of water and co-solute ions and/or molecules for the decrease in solvent-excluded volume associated with polypeptide folding (i.e., there is a large decrease in solvent-accessible surface area). The magnitude of this entropic contribution increases with the number density and volume packing density of the solution. The two destabilizing contributions come from the conformational entropy of the chain, which should not depend significantly on the presence of co-solutes, and from the direct energetic interactions between co-solutes and the protein surface in both the native and denatured states. It is the magnitude of the latter that discriminates between stabilizing and destabilizing agents.

## 1. Introduction

It is well established that the conformational stability of globular proteins can be markedly modified by the addition of small co-solutes, both neutral and charged, to water, which can be either stabilizing or destabilizing to the native state. Even though a huge number of investigations, including experimental, computational, and theoretical, have been performed, a complete molecular-level understanding is still lacking [1,2,3,4,5,6,7,8,9,10]. The common view is that stabilizing agents are preferentially excluded from the protein solvation shell, whereas destabilizing agents are preferentially accumulated in the protein solvation shell. It should be recognized that preferential exclusion or preferential accumulation are thermodynamic concepts, which do not provide an actual molecular mechanism [4].

Forty years ago, Arakawa and Timasheff reported that the addition of sodium glutamate (NaGlu) increased the thermal stability of hen egg white lysozyme and bovine serum albumin [11], causing a preferential hydration of the proteins. They interpreted these results in terms of an unfavorable Gibbs free energy of interaction of NaGlu with protein surfaces. This interesting finding was not followed by other experimental investigations until Record, Rayleigh, and co-workers [12] conducted a careful study of the stabilization provided by potassium glutamate (KGlu) to the N-terminal domain of the ribosomal protein L9, and concluded that KGlu has a strong stabilizing action towards the folded state. According to the approach developed by Record, the KGlu stabilization originated from the unfavorable interactions of KGlu with both the hydrocarbon surface area and the amide surface area [13]; this proved to be an unusual situation, but a molecular-level explanation of such unfavorability was not provided. It should be noted that Record’s approach is grounded on the additivity principle of energetic interactions, assuming the protein surface can be divided in two parts: a nonpolar hydrocarbon one and a polar amide one. This additivity principle cannot be taken for granted in the case of heteropolymers [14], such as globular proteins, consisting of different chemical groups, some of which are charged and more or less randomly dispersed on the surface, and some of which are able to make H-bonds with water molecules. Moreover, additivity should not hold in aqueous solutions containing ions such as aspartate and glutamate, which, having three charges at pH 7.0 (two negative and one positive on the basis of the three pKa values), localized in different parts of the chemical structure, are involved in long-range Coulomb interactions (both attractive and repulsive).

In order to add something new and interesting to the puzzle, we decided to study the effect of both KAsp and KGlu on the conformational stability of a model globular protein, such as RNase A, by performing differential scanning calorimetry (DSC) measurements. The rationale was as follows: (a) to directly measure the denaturation enthalpy and entropy changes that build up the denaturation Gibbs free energy change and (b) to verify if aspartate is a stabilizing agent, like glutamate, a test that has not been conducted yet to the best of our knowledge. Moreover, we studied the counteraction of the stabilization afforded by KAsp and KGlu caused by the addition of denaturing agents, such as urea, guanidinium chloride (GdmCl), and guanidinium thiocyanate (GdmSCN), always performing DSC measurements. Experimental data are analyzed and discussed on the basis of a theoretical approach developed by one of the authors.

## 2. DSC Results

**Figure 1:**
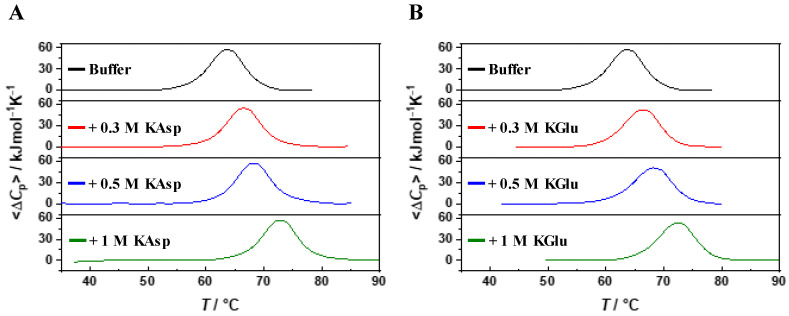
DSC profiles of RNAse A in 10 mM MOPS + 100 mM NaCl buffer, pH 7.0, in the absence and presence of KAsp (panel **A**) or KGlu (panel **B**) at the indicated concentrations.

DSC measurements, shown in Figure 1, indicate that the temperature-induced denaturation of RNase A in 10 mM MOPS buffer with 100 mM NaCl, pH 7.0, is a reversible two-state process in all the considered aqueous solutions (i.e., the so-called re-heating criterion has been used to test the reversibility; the closeness to one of the values of the calorimetric to van’t Hoff enthalpy ratio, usually indicated as cooperative unit (CU) has been used to test the two-state cooperativity, see the fifth column of Table 1).

The T_d_ values listed in the second column of Table 1 demonstrate that the protein thermal stability increases significantly upon increasing the concentration of both KAsp and KGlu: T_d_ passes from 63.5 °C in aqueous buffer solution to 72.7 °C in 1 M KAsp and to 72.3 °C in 1 M KGlu. These values imply that potassium aspartate and potassium glutamate are strong stabilizing agents of the folded state and their effects are closely similar; indeed, we found that T_d_ = 66.2 °C in 1 M glucose, 67.2 °C in 1 M betaine, 67.6 °C in 1 M sucrose, 67.8 °C in 1 M TMAO, and 68.4 °C in 1 M trehalose [15,16]. It is worth underscoring that (a) such a comparison is somewhat misleading because KAsp and KGlu are salts that dissociate in water, producing two ions, so that the actual molar concentration of co-solutes is larger than 1 M, and (b) the presence of two ion types, one of which contains three charges, implies that long-range Coulomb interactions are operative.

**Figure 2:**
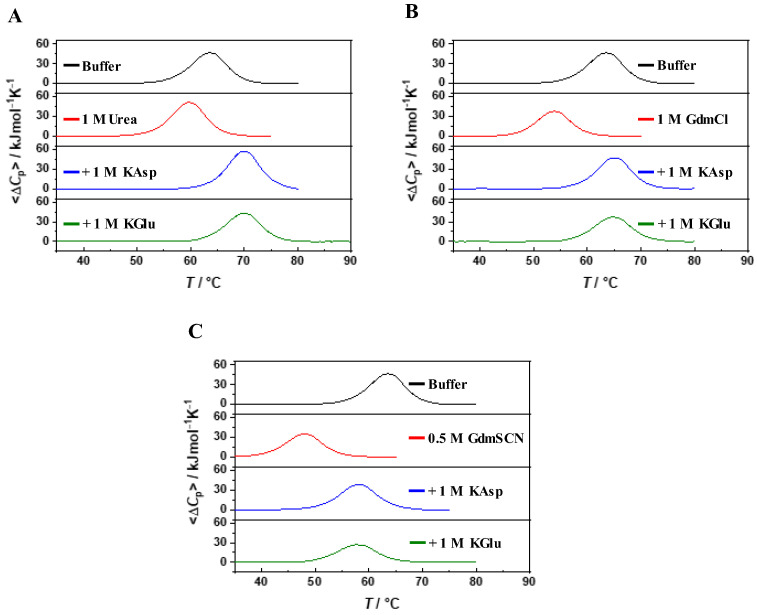
DSC profiles of RNAse A in aqueous buffer, 1 M urea, 1 M KAsp plus 1 M urea, and 1 M KGlu plus 1 M urea (panel **A**); DSC profiles of RNAse A in aqueous buffer, 1 M GdmCl, 1 M KAsp plus 1 M GdmCl, and 1 M KGlu plus 1 M GdmCl (panel **B**); DSC profiles of RNAse A in aqueous buffer, 0.5 M GdmSCN, 1 M KAsp plus 0.5 M GdmSCN, and 1 M KGlu plus 0.5 M GdmSCN (panel **C**).

As shown in Figure 2, the addition of denaturing agents, such as urea, GdmCl, and GdmSCN, counteracts the stabilizing effect of KAsp and KGlu; for instance: (a) T_d_ = 64.9 °C in 1 M KAsp + 1 M GdmCl, and 64.7 °C in 1 M KGlu + 1 M GdmCl; (b) T_d_ = 58.0 °C in 1 M KAsp + 0.5 M GdmSCN, and 57.9 °C in 1 M KGlu + 0.5 M GdmSCN. These results confirm that counteraction is a general phenomenon independent of the chemical nature and structure of the stabilizing and destabilizing agents [15,16,17].

The values of the denaturation enthalpy change, ΔH_d_(T_d_), listed in the third column of Table 1, indicate the following: (a) KAsp and KGlu, notwithstanding their marked ability to raise the T_d_ value, do not cause a significant ΔH_d_(T_d_) increase; this is a strong indication that the stabilization provided by them has an entropic origin. (b) The addition of the denaturing agents, in particular GdmCl or GdmSCN, causes a significant ΔH_d_(T_d_) decrease. The present DSC data do not produce a linear plot of ΔH_d_(T_d_) versus T_d_, so it is not possible to obtain an estimate of ΔC_p,d_. However, the latter is needed to calculate the denaturation Gibbs free energy at 25 °C in all the considered aqueous solutions, starting from the experimental T_d_ and ΔH_d_(T_d_) values, by means of the Gibbs–Helmholtz equation [18]. In a previous work, we obtained ΔC_p,d_ = 6.4 ± 0.7 kJ K^−1^mol^−1^ from a ΔH_d_(T_d_) versus T_d_ plot, consisting of 45 points coming from our own DSC measurements of RNase A in different aqueous solutions [16]. The latter ΔC_p,d_ value has been used to calculate the ΔG_d_(25 °C) estimates reported in the fifth column of Table 1; the trend of these estimates is in line with that of the experimental T_d_ values, even though the significance of the latter is simpler to grasp.

These experimental data need a reliable rationalization that has to come from a theoretical approach based on statistical mechanics.

## 3. Theoretical Approach and Its Application

It has been shown by one of the authors that the conformational stability of globular proteins in aqueous solutions can be rationalized by means of a theoretical approach, grounded on the assumption that polypeptide chains can populate two macrostates, the native one (N-state) and the denatured one (D-state). The approach singles out three fundamental contributions to the denaturation Gibbs free energy change (ΔG_d_), as shown by the following equation [15,16,17,19,20,21]:

ΔG_d_ = ΔΔGc − TΔS_conf_ + ΔE_a_ (1)

Now, the physico-chemical meaning of the three terms will be spelled out in detail. The first term on the right-hand side of Equation (1), ΔΔGc, represents the loss in translational entropy of solvent molecules associated with the increase in solvent-excluded volume upon denaturation. It is necessary to provide an explanation of what the solvent-excluded volume effect is to avoid misunderstandings. In a condensed state of matter, such as a liquid, the space for the insertion of a solute molecule has to be created: the void space is a large fraction of the total volume, but it is partitioned in very small pieces (as a consequence of the size of liquid molecules) which prove to be not available for the insertion of a molecule. This means that reversible work has to be carried out for cavity creation. The latter is a special process because it cannot be studied from the experimental point of view, but solely by means of theoretical approaches and computer simulations. The creation of a cavity (assumed to be spherical for simplicity) at a fixed position in a liquid, keeping constant temperature and pressure, causes an increase in the liquid volume equal to the van der Waals volume of the cavity itself. However, the cavity’s existence produces a geometric constraint for the liquid molecules (both solvent and co-solute): the centers of the latter cannot enter the spherical shell between the van der Waals surface of the cavity and its solvent-accessible surface area; otherwise, the cavity would not exist, because its volume would be occupied. This geometric constraint produces a solvent-excluded volume effect for all the molecules or ions in the liquid system that are in continuous motion. This solvent-excluded volume effect leads to a significant decrease in the total number of available spatial configurations, and so in a loss of translational entropy of solvent and co-solute molecules. This reasoning implies that the magnitude of the solvent-excluded volume effect is proportional to the solvent-accessible surface area of the cavity (i.e., of the inserted solute molecule). As a consequence, in the case of globular protein denaturation, this entropic contribution largely favors the N-state (i.e., water molecules push the polypeptide chain to collapse in order to gain translational entropy).

The entropic contribution due to the solvent-excluded volume effect can be calculated as the difference in the reversible work to create a cavity suitable to host the D-state and a cavity suitable to host the N-state:

ΔΔGc = ΔGc(D-state) − ΔGc(N-state)(2)

Reliable ΔGc values can be calculated by means of the analytical relationship provided by classic scaled particle theory [22,23] (SPT), assuming that simple geometric models can be used to represent the two protein macrostates. Specifically, a sphere represents the N-state and a prolate spherocylinder represents the D-state, with a fundamental constraint: the sphere and the prolate spherocylinder must have the same van der Waals volume. Experimental measurements indicate, indeed, that the volume change associated with denaturation is a negligibly small quantity [24,25,26]. Clearly, even though the two objects have the same van der Waals volume, their water-accessible surface area (WASA) [27] is different (as their solvent-excluded volume):

WASA(prolate spherocylinder) > WASA(sphere)(3)

This is in line with experimental findings indicating that [28,29]:

WASA(D-state) > WASA(N-state)(4)

According to previous choices [15,16,17,21], the geometric features of the two objects are the following: considering that the model protein has 138 residues and the average van der Waals volume of a residue in the protein interior is 102.5 Å^3^ [30], its V_vdW_ = 14,145 Å^3^; the sphere representing the N-state has radius *a* = 15 Å and WASA = 3380 Å^2^ (note that, to calculate WASA, the radius assigned to water molecules is 1.4 Å, the customary one [27]); finally, the prolate spherocylinder representing the D-state has radius *a* = 6 Å, cylindrical length *l* = 117 Å, and WASA = 6128 Å^2^. The ΔGc values have been calculated by means of the following analytical relationship provided by classic SPT for a prolate spherocylindrical cavity of radius *a* and cylindrical length *l* in a hard sphere fluid mixture (the pressure–volume term is neglected for its smallness at P = 1 atm) [20]:

ΔGc = RT·{−ln(1 − ξ_3_) + [6ξ_2_/(1 − ξ_3_)]*a* + [12ξ_1_/(1 − ξ_3_)]*a*^2^ + [18ξ_2_^2^/(1 − ξ_3_)^2^]*a*^2^ + [3ξ_2_/2(1 − ξ_3_)]*l* + [6ξ_1_/(1 − ξ_3_)]*a·l* + [9ξ_2_^2^/(1 − ξ_3_)^2^]*a·l*} (5)

where ξ_i_ = (π/6)·Σρ_j_·σ_j_^i^, and ρ_j_ is the number density, in molecules per Å^3^, of the species j and σ_j_ is the corresponding hard sphere diameter; ξ_3_ = (π/6)⋅∑ρ_j_⋅σ_j_^3^ represents the volume packing density of the hard sphere fluid mixture (i.e., the fraction of the total volume really occupied by solvent molecules and co-solute ions and molecules). By setting *l* = 0, the formula is that for a spherical cavity of radius *a*; by considering only one component, Equation (5) is that for a hard sphere fluid.

Experimental values of the density of water [31] and all the considered aqueous salt solutions (measured by us), at 25 °C and 1 atm, have been used to perform calculations. The selected effective hard sphere diameters are as follows: σ = 2.80 Å for water molecules [32,33]; 4.64 Å for urea molecules and 4.70 Å for Gdm^+^ ions (these two correspond to the diameter of the sphere possessing the same WASA of the molecule or ion [15]); 6.06 Å for Asp^−^ ions and 6.37 Å for Glu^−^ ions (these two correspond to the diameter of the sphere obtained by summing up the volume group contributions devised by Ben–Amotz and Willis [34]); 2.66 Å for K^+^ ions and 3.62 Å for Cl^−^ ions (these come from the ionic radii fixed by Pauling [35]); and 3.94 Å for SCN^−^ ions, the same hard sphere diameter of carbon dioxide [15]. All these values are listed in the fourth column of Table 2.

Classic SPT is a hard sphere theory and can solely account for the effect that co-solute addition has on the number density of the liquid phase, and so on the magnitude of the solvent-excluded volume effect. On the other hand, it is well established that charged co-solutes, in view of the strength of their electric fields, markedly affect the tetrahedral structure of water, and so the translational and rotational entropy of water molecules [36]. These important aspects cannot be accounted for by the present approach.

The second term on the right-hand side of Equation (1), T·ΔS_conf_, represents the gain in conformational entropy of the polypeptide chain upon denaturation. It is straightforward to guess the sign of this entropic term, but it is very difficult to make a correct calculation. The N-state represents an ensemble of conformations confined in a small volume of the phase space, whereas the D-state represents a huge ensemble of fluctuating conformations occupying a significant fraction of the phase space. This implies that the conformational entropy change upon denaturation is a large positive quantity, favoring the stability of the D-state (i.e., there is a large gain in conformational degrees of freedom for the polypeptide chain upon denaturation). A reliable evaluation of this contribution requires the availability of realistic ensembles for the two macrostates, generated by means of large-scale computer simulations. Such a task has been undertaken by some research groups, and it is possible to take advantage of their results [37,38,39]. Notwithstanding the complexity of the situation [40], it appears to not be wrong to assume that each residue gives the same independent contribution, ΔS_conf_(res) = 19 J K^−1^molres^−1^ [21,37]. The latter value, using the well-known Boltzmann formula, leads to Ω(D-state)/Ω(N-state) = 9.8, where Ω is the number of rotameric states accessible to an average residue. Thus, the overall conformational entropy term can be calculated as follows:

T·ΔS_conf_ = T·N_res_·ΔS_conf_(res) (6)

It is interesting to note that Rose [41], by means of a simple calculation based on the allowed surface area in the Ramachandran map, obtained ΔS_conf_(res) = 17.7 J K^−1^molres^−1^, which is close to the value reported above. The magnitude of the conformational entropy gain upon denaturation should depend little on the presence of co-solutes in the aqueous solution because these ions and molecules have weak interactions with proteins (in both the N-state and the D-state) due to the ubiquitous competition with water molecules. This reasoning implies that the conformational features of the two macrostates are not modified, and the T·ΔS_conf_ term can be considered to not be affected by the presence of co-solutes [15,16,17].

The third term on the right-hand side of Equation (1), ΔE_a_, represents the contribution accounting for the energetic interactions of the N-state and D-state with water molecules and co-solute ions and molecules and the difference in intra-protein interactions between the two macrostates. This energetic term is given by the following equation:

ΔE_a_ = E_a_(D-state-water) − E_a_(N-state-water) + ΔE(intra) (7)

In aqueous solutions not containing co-solutes, the ΔE_a_ values should be close to zero because an almost perfect balance for the energetic interactions between the N-state and the D-state is operative [21] (for instance, the gain in protein–water H-bonds upon denaturation corresponds to the loss of intra-protein H-bonds, in particular for the unfolding of secondary structure elements [42]). Fixing ΔE_a_ = 0, and calculating the other two terms constituting the denaturation Gibbs free energy (see the first line in Table 3), it results that ΔG_d_ = ΔΔGc − TΔS_conf_ = 803 − 782 = 21 kJ mol^−1^ at 25 °C and 1 atm. The N-state of our model protein is more stable than the D-state by a small quantity (i.e., it is the balance of two great and contrasting contributions), in line with its marginal stability [18,19,20,21]. The calculated ΔG_d_ value is not far from that obtained by means of DSC measurement for RNase A at pH 7.0 (see the first number in the last column of Table 1). In general, it is reliable for a globular protein, because it corresponds to 152 J mol^−1^ per residue [43]. These comparisons indicate that the present “geometric” approach is effective.

In the presence of co-solutes, the ΔE_a_ values are negative, and their magnitude proves to be large in the case of denaturing agents [15,16,17]. The latter interact more favorably with protein surfaces than with water molecules, and tend to accumulate in the protein solvation shell, favoring denaturation. The ΔE_a_ magnitude can become so large upon increasing the concentration of the denaturing agent that it also causes denaturation around room temperature [44]. Reliable estimates of the ΔE_a_ contribution are not simple to achieve because it is necessary to generate reliable conformational ensembles for the two macrostates and to have good force fields to describe all the feasible water–water, water–protein, water–co-solute, and protein–co-solute interactions [45,46].

The present theoretical approach does not lead to a direct relationship allowing one to calculate the value of the denaturation temperature; rather, it allows the calculation of ΔG_d_, and one should find out the temperature at which ΔG_d_ = 0 to obtain an estimate of T_d_. Even though this is a feasible task, the uncertainties are large because one has to account for the temperature dependence of several quantities [21]. Nevertheless, it is useful to apply this theoretical approach to the investigated binary and ternary aqueous solutions at room temperature. The ΔGc values for both the N-state and the D-state, in all the considered aqueous solutions at 25 °C, have been calculated by means of Equation (5), and are listed in the second and third columns of Table 3. The corresponding ΔΔGc = ΔGc(D-state) − ΔGc(N-state) values, reported in the fourth column of Table 3, are large and positive, indicating that the decrease in solvent-excluded volume associated with protein folding (i.e., the shape change from the prolate spherocylinder to the sphere, keeping fixed the van der Waals volume) is the main stabilizing contribution of the N-state (i.e., this contribution represents most of what is usually called the hydrophobic effect) [20,47,48,49]. Moreover, the ΔΔGc′ = ΔΔGc(other) − ΔΔGc(water) values, listed in the last column of Table 3, are all positive, indicating that (a) the addition of all the considered co-solutes leads to an increase in the ΔGc magnitude and (b) it is not the ΔΔGc′ sign that can discriminate between stabilizing and destabilizing agents. On the other hand, it is evident that the magnitude of ΔΔGc′ is significantly larger in the case of (a) the two stabilizing agents, KAsp and KGlu (i.e., ΔΔGc′ = 96 kJ mol^−1^ in 1 M KAsp, and 107 kJ mol^−1^ in 1 M KGlu), and (b) all the ternary aqueous solutions (i.e., ΔΔGc′ = 145 kJ mol^−1^ in 1 M KAsp + 1 M GdmCl and 185 kJ mol^−1^ in 1 M KGlu + 1 M GdmCl). An explanation is provided by the numbers listed in the second column of Table 2, which allow the calculation of the molar density of such aqueous solutions, and by the numbers listed in the fifth column of Table 2, which represent the volume packing density of each solution. The result is that the magnitude of ΔΔGc′ is large when (a) the overall molar density is not far from that of water (which has the largest molar density among all common liquids and solutions [15,16,17]) and (b) the volume packing density is markedly larger than that of water (which has a small ξ_3_ value as a consequence of the small size of its molecules and its tetrahedral coordination). The latter two variables determine the magnitude of the solvent-excluded volume effect, which, in turn, determines the magnitude of ΔGc.

Moreover, since the T·ΔS_conf_ term has been assumed to be independent of the presence of co-solutes, the further distinction between stabilizing and destabilizing agents has to come from the ΔE_a_ term. The latter is expected to be a negative quantity in all cases; however, its magnitude should be large only in the case of destabilizing agents, as a consequence of favorable energetic attractions to protein surfaces (i.e., the qualitative difference between buried and accessible surfaces of globular proteins is smaller than imagined; according to the analysis by Harpaz, Gerstein, and Chothia, on average, the buried surface is 62% nonpolar, 31% polar, and 7% charged, whereas the accessible surface is 56% nonpolar, 27% polar, and 17% charged [50]) made by urea molecules, Gdm^+^, and SCN^−^ ions [51,52].

The occurrence of such favorable energetic attractions is strongly supported by the large number of “binding” sites for the above species found in several protein structures deposited in the Protein Data Bank and carefully characterized [53,54,55]. In particular, the guanidinium and thiocyanate ions prove to be “sticky” [56], because their low charge density and large polarizability due to π-electrons allow the formation of good dispersion attractions with both polar and nonpolar moieties. In contrast, the aspartate and glutamate ions possess two negative charges and one positive charge at pH 7.0, localized in different parts of the chemical structure, that favor the formation of strong H-bonds with water molecules. This implies that aspartate and glutamate prefer to stay in water with respect to interacting with protein surfaces, in line with the preferential hydration measured by Arakawa and Timasheff [11]. Indeed, the molar density and volume packing density of KAsp and KGlu aqueous solutions are large enough to cause a significant ΔGc increase that leads to a marked stabilization of the N-state. A simple analysis may be useful. On recognizing that the van der Waals volume of Asp^−^ and Glu^−^ is around 10 times larger than that of water (look at the diameter values listed in the fourth column of Table 2 and note that the potassium ion has a diameter slightly smaller than that of a water molecule), the conclusion should be that their presence causes a significant decrease in water molar density. Such a decrease, however, does not happen (i.e., [H_2_O] = 55.3 M in pure water, 50.7 M in 1 M KAsp, and 50.1 M in 1 M KGlu), because of the several strong H-bonds established between Asp^−^ and Glu^−^ and water molecules. This is a clear manifestation of the strong electrostriction caused by such ions [57,58]. In the same vein, in the considered ternary aqueous solutions, the ΔΔGc′ values prove to be very large and positive and can only be partially offset by the large negative ΔE_a_ terms provided by the presence of 1 M urea, 1 M GdmCl, or 0.5 M GdmSCN; the consequence is that the T_d_ values of RNase A remain high (see the second column of Table 1).

## 4. Materials and Methods

### 4.1. Materials

Pancreatic ribonuclease, RNase A (Type XII-A), urea, GdmCl, GdmSCN, Kasp, and KGlu were from Merck (Darmstadt, Germany). A buffer solution of 10 mM MOPS (3-(N-morpholino) propanesulfonic acid) with 100 mM NaCl at pH 7.0 was used. MOPS buffer was chosen for its low protonation enthalpy to reduce the temperature dependence of the solution pH. Chemicals for buffering were of analytical grade from Sigma and dissolved in Milli Q (Millipore, Bedford, MA, USA) water. Urea stock solutions were prepared, just before use, by weight in a 3 mL final volume calibrated flask. GdmCl and GdmSCN were purchased as ready-to-use 8 M and 6 M buffered solutions, respectively. Protein stock solutions were extensively dialyzed against the buffer; the RNase A concentration was determined by UV spectra, using a sequence-based extinction coefficient of 9440 M^−1^cm^−1^ at 280 nm [59]. Samples for DSC measurements were prepared by mixing appropriate volumes of the protein stock solution with co-solute stock solutions, then diluting with buffer up to a fixed volume of 2 mL in a calibrated flask. The final protein concentration was kept constant at 1 mg mL^−1^.

### 4.2. Density Measurements

The density of binary (containing water and KAsp or KGlu) and ternary (containing water, KAsp or KGlu, and a denaturant) solutions was measured using a vibrating tube densimeter (Anton Paar 5000, North Ryde, Austria), which has an accuracy of 0.5 g dm^−3^, at a constant cell temperature of 25.000 ± 0.001 °C. Samples were prepared and measured in triplicate. For the preparation, a 2 mL calibrated flask was employed; the required amount of co-solute(s) was taken by weight, and then water was added until reaching the final volume.

### 4.3. Differential Scanning Calorimetry (DSC)

DSC measurements were performed on a Nano-DSC (TA Instruments, New Castle, DE, USA), which has an active cell volume of 0.3 mL and works at a pressure of 3 atm. The scan speed was set at 1 °C min^−1^. Samples were prepared in a 3 mL calibrated flask by adding the appropriate amount of protein and co-solute(s) and diluting with buffer until reaching the final volume. Each measurement was conducted in triplicate, measuring the appropriate blank (buffer or buffer plus co-solute) before the sample, and two scans were performed on each sample to check the reversibility of the thermal denaturation. The data were analyzed using the Nano-Analyze software 3.12.0 supplied by the manufacturer. The blank scan was subtracted by the sample scan. The excess heat capacity, <ΔC_p_>, was obtained by taking as baseline the linear temperature dependence of the heat capacity of the native state [60]. The calorimetric enthalpy change, ΔH_d_(T_d_), was obtained by integrating the area under the curve. Using the Nano-Analyze software, the experimental DSC curves were modelled according to the two-state reversible equilibrium to calculate the van’t Hoff enthalpy change, ΔH_d_^vH^(T_d_), and the cooperative unit (CU), defined as the calorimetric-to-van’t Hoff enthalpy ratio. A CU value close to 1 is a necessary condition to state that the observed transition is a cooperative two-state process [61,62]. The Gibbs free energy of denaturation at 25 °C, ΔG_d_(25 °C), was calculated via the Gibbs–Helmholtz equation using the T_d_, ΔH_d_(T_d_) and ΔC_p,d_ values obtained from the measurements.

## 5. Conclusions

We have shown that KAsp and KGlu significantly stabilize the native state of RNase A with respect to temperature-induced denaturation. This stabilization is counteracted by the addition of common denaturants such as urea, GdmCl, and GdmSCN. A rationalization of the experimental data, albeit with approximations, is provided by means of a theoretical approach.

## Figures and Tables

**Table 1:** Thermodynamic parameters from the analysis of DSC curves for the temperature-induced denaturation of RNase A in the presence of different concentrations of KAsp and KGlu and in the presence of 1 M concentration of one of the previous salts and some denaturing agents. CU, cooperative unit, is defined as the calorimetric-to-van’t Hoff enthalpy ratio (see Section 4: Materials and Methods).

	T_d/_°C [a]	ΔH_d_ (T_d_)/kJ mol^−1^ [a]	CU	ΔG_d_ (25 °C)/kJ mol^−1^
H_2_O	63.5	464	0.99	38
0.3 M KAsp	66.4	455	0.99	39
0.5 M KAsp	68.2	480	0.97	42
1 M KAsp	72.7	482	0.99	44
0.3 M KGlu	66.3	485	0.97	42
0.5 M KGlu	68.2	455	0.99	39
1 M KGlu	72.2	480	0.97	44
1 M urea	59.7	440	1.0	34
1 M KAsp + 1 M urea	69.9	460	0.99	41
1 M KGlu + 1 M urea	68.8	420	0.98	35
1 M GdmCl	53.9	383	0.97	25
1 M KAsp + 1 M GdmCl	64.9	400	0.99	31
1 M KGlu + 1 M GdmCl	64.7	378	0.98	29
0.5 M GdmSCN	47.9	320	0.96	17
1 M KAsp + 0.5 M GdmSCN	58.0	352	0.96	24
1 M KGlu + 0.5 M GdmSCN	57.9	360	0.97	25

[a] The experimental errors for T_d_ and ∆H_d_(T_d_) are within 0.2 °C and 5%, respectively, of the reported values. Each number represents the mean value of at least three DSC measurements.

**Table 2:** Experimental values of the density and water molar concentration for pure water, and for considered binary and ternary aqueous solutions at 25 °C and 1 atm; values of the effective hard sphere diameters, σ assigned to all species; values of the volume packing density, ξ_3_ for the solutions; values of the average effective hard sphere diameter, <σ> = ∑χ_j_·σ_j_, where χ_j_ is the molar fraction of the species j and σ_j_ is the corresponding hard sphere diameter.

	ρ/g L^−1^	[H_2_O]/M	σ/Å	ξ_3_	<σ>/Å
H_2_O	997	55.3	2.80	0.383	2.80
1 M KAsp	1085	50.7	6.06 and 2.66	0.427	2.86
1 M K Glu	1088	50.1	6.37 and 2.66	0.434	2.87
1 M urea	1013	52.9	4.64	0.398	2.83
1 M KAsp + 1 M urea	1099	48.2	6.06 and 2.66 and 4.64	0.441	2.90
1 M KGlu + 1 M urea	1104	47.7	6.37 and 2.66 and 4.64	0.449	2.90
1 M GdmCl	1024	51.5	4.70 and 3.62	0.404	2.85
1 M KAsp + 1 M GdmCl	1109	46.7	6.06 and 2.66 and 4.70 and 3.62	0.447	2.91
1 M KGlu + 1 M GdmCl	1112	46.1	6.37 and 2.66 and 4.70 and 3.62	0.458	2.92
0.5 M GdmSCN	1011	52.8	4.70 and 3.94	0.392	2.83
1 M KAsp + 0.5 M GdmSCN	1095	48.0	6.06 and 2.66 and 4.70 and 3.94	0.434	2.89
1 M KGlu + 0.5 M GdmSCN	1101	47.5	6.37 and 2.66 and 4.70 and 3.62	0.442	2.90

**Table 3:** Classic SPT estimates of the reversible work to create, in the reported aqueous solutions at 25 °C and 1 atm, cavities corresponding to the N-state (i.e., a sphere of 15 Å radius) and to the D-state (i.e., a prolate spherocylinder of 6 Å radius and 117 Å cylindrical length), respectively; values of ΔΔGc’ = ΔΔGc(co-solute) − ΔΔGc(water). All the numbers are in kJ mol^−1^ units; see text for further details.

	ΔGc(N)	ΔGc(D)	ΔΔGc	ΔΔGc^’^
H_2_O	1072	1875	803	-
1 M urea	1111	1942	831	28
1 M GdmCl	1135	1984	849	46
0.5 M GdmSCN	1085	1897	812	9
1 M KAsp	1203	2102	899	96
1 M KAsp + 1 M urea	1245	2176	931	128
1 M KAsp + 1 M GdmCl	1269	2217	948	145
1 M KAsp + 0.5 M GdmSCN	1207	2109	902	99
1 M KGlu	1218	2128	910	107
1 M KGlu + 1 M urea	1268	2215	947	144
1 M KGlu + 1 M GdmCl	1323	2311	988	185
1 M KGlu + 0.5 M GdmSCN	1228	2147	919	116

## Data Availability

Dataset available on request from the authors.
